# Developmental Changes of BOLD Signal Correlations with Global Human EEG Power and Synchronization during Working Memory

**DOI:** 10.1371/journal.pone.0039447

**Published:** 2012-07-06

**Authors:** Lars Michels, Rafael Lüchinger, Thomas Koenig, Ernst Martin, Daniel Brandeis

**Affiliations:** 1 Center for MR-Research, University Children’s Hospital, Zurich, Switzerland; 2 Institute of Neuroradiology, University Hospital of Zurich, Zurich, Switzerland; 3 Department of Child and Adolescent Psychiatry, University of Zürich, Zurich, Switzerland; 4 Department of Psychiatric Neurophysiology, University Hospital of Psychiatry, Bern, Switzerland; 5 Zurich Center for Integrative Human Physiology, University of Zurich, Zurich, Switzerland; 6 Department of Child and Adolescent Psychiatry and Psychotherapy, Central Institute of Mental Health, Medical Faculty Mannheim/Heidelberg University, Heidelberg, Germany; University Medical Center Groningen UMCG, Netherlands

## Abstract

In humans, theta band (5–7 Hz) power typically increases when performing cognitively demanding working memory (WM) tasks, and simultaneous EEG-fMRI recordings have revealed an inverse relationship between theta power and the BOLD (blood oxygen level dependent) signal in the default mode network during WM. However, synchronization also plays a fundamental role in cognitive processing, and the level of theta and higher frequency band synchronization is modulated during WM. Yet, little is known about the link between BOLD, EEG power, and EEG synchronization during WM, and how these measures develop with human brain maturation or relate to behavioral changes. We examined EEG-BOLD signal correlations from 18 young adults and 15 school-aged children for age-dependent effects during a load-modulated Sternberg WM task. Frontal load (in-)dependent EEG theta power was significantly enhanced in children compared to adults, while adults showed stronger fMRI load effects. Children demonstrated a stronger negative correlation between global theta power and the BOLD signal in the default mode network relative to adults. Therefore, we conclude that theta power mediates the suppression of a task-irrelevant network. We further conclude that children suppress this network even more than adults, probably from an increased level of task-preparedness to compensate for not fully mature cognitive functions, reflected in lower response accuracy and increased reaction time. In contrast to power, correlations between instantaneous theta global field synchronization and the BOLD signal were exclusively positive in both age groups but only significant in adults in the frontal-parietal and posterior cingulate cortices. Furthermore, theta synchronization was weaker in children and was –in contrast to EEG power– positively correlated with response accuracy in both age groups. In summary we conclude that theta EEG-BOLD signal correlations differ between spectral power and synchronization and that these opposite correlations with different distributions undergo similar and significant neuronal developments with brain maturation.

## Introduction

Simultaneous resting-state functional magnetic resonance imaging (fMRI) and electroencephalography (EEG) studies in adults have revealed that theta (4–7 Hz) and alpha (8–13 Hz) band power are inversely related to the fMRI (i.e., blood oxygen level dependent, BOLD) signal [1], [2], [3], [4]. During short-term working memory (WM), one of the best replicated findings is that theta band power increases with load in frontal midline regions, seen as FM-theta [5], [6], [7]. Simultaneous EEG-fMRI recordings have revealed negative theta-BOLD signal correlations during mental arithmetic [8], [9] and during the maintenance interval of a short-term WM task [10], [11], which anatomically overlap with regions of the default mode network (DMN). The DMN is believed to consist of a set of brain regions, including cingulate, prefrontal and parietal regions, which consistently show decreased neuronal activity during goal-oriented tasks [12], [13]. Functional brain changes related to maturation have been found within and outside the DMN. For example, resting-state fMRI studies revealed that long range connections of the DMN, especially along the anterior-posterior axis, such as the connection from the anterior to the posterior cingulate cortex, are not fully established in children [14], [15]. Further, neurodevelopmental fMRI investigations generally report greater recruitment of brain regions involved in WM in adults than in children [16], [17], especially within the posterior parietal cortex (PPC), Broca’s area, dorsolateral lateral prefrontal cortex (DLPFC), and medial prefrontal cortex (MPFC). In addition to fMRI studies, neuroelectrical studies have identified markers for brain immaturity. Specifically, it has been shown by resting-state EEG studies that low frequency power, including theta, is elevated in children compared to adults [18], [19], [20]. However, direct comparisons between memory related theta activity in children and adults are rare [21], [22], [23].

While EEG band power reflects an important physiological correlate of information processing, it is insensitive to the degree of neuronal synchronization among brain signals. One putative mechanism for cortical differentiation and large scale integration of widely distributed areas of the brain is the formation of dynamic links mediated by neuronal synchrony [24], [25], [26], [27], [28]. Synchronization across space (i.e., not across spectral frequencies) is a complementary aspect of brain electrical activity and has been suggested to represent a mechanism for organizing brain function [27], [29]. During rest, global field synchronization (GFS) is not stable but fluctuates in frequency and appears as transient intervals during which similar signals are observed simultaneously across all electrodes [30]. Several studies have demonstrated that synchronization is modulated during cognitive processing in theta and other frequency bands [31], [32], [33], [34], [35], and additionally increases during brain development [22], [36]. It has also been demonstrated that task-related (i.e., during auditory-oddball performance) theta [22] and alpha [37], [38], [39] EEG phase synchronization increases from childhood to adulthood, while EEG power decreases. Although these studies suggest that those EEG metrics measured during a task capture developmental effects, it is unclear whether synchronicity patterns and power in adults and children are coupled to the BOLD signal during cognitive processing. Thus, it has not yet been investigated whether EEG-BOLD signal coupling strength is affected by age-related task performance.

In this study, we focused on the maintenance interval of a load-modulated Sternberg WM task [40]. This task has been used in adults [41], [42], but variants of the Sternberg task have also been used in developmental fMRI studies [16], [43] or other WM studies [17], [44], [45], [46], [47], [48], [49]. We tested two hypotheses regarding developmental changes of EEG-BOLD correlations: First, we hypothesized that theta power- and theta synchronization-BOLD signal correlations would reveal developmental effects. Children’s theta-BOLD signal correlations were assumed to be partly manifested in the same network of brain regions as in adults (i.e., the DMN) but with a different strength, and to partly correlate in a different network of brain regions. Additionally, we hypothesized that children and adults might show statistically non-differentiable theta-BOLD signal coupling patterns, if the physiological coupling matures earlier and is insensitive to the developmental cognitive effects which are seen with EEG and fMRI alone. Apart from these hypotheses, in adults we expected to replicate the negative correlation of theta power with the BOLD signal [10], [11].

## Materials and Methods

### Participants

18 adults (mean age 24.9±3.8 years, 8 males) and 15 children (mean age 10.2±1.3 years, 10 boys), all right-handed and normal sighted, were included in this study. Resting-state data from a nearly identical group of subjects have been already presented in a previous EEG-fMRI paper from our group [50]. All participants met the MRI safety standards, were healthy, i.e. with no history of medical or psychiatric disease, and were not currently taking drugs or medication. All participants as well as the parents/caregivers of the children gave written informed consent prior to participation. This study is approved by the ethical committee (‘Kantonale Ethikkommission Zürich’, http://www.kek.zh.ch, StV 08/08).

### Task and Procedure

Participants performed a workload-modulated Sternberg WM task [40] that temporally separates encoding, maintenance, and retrieval processes [10]. At the onset of each of the 64 trials, an array of 15 items appeared in three rows (encoding interval). The stimulus (2.5 s) consisted of sets of either 2 or 5 consonants (load 2 and load 5 conditions). The remaining items were plus signs (+). The position of the consonants was counterbalanced across trials. The stimulus array was followed by maintenance interval of 3.5 s, during which a central fixation cross was presented. After the maintenance interval, a probe letter was shown for 2 s (retrieval interval). The inter-stimulus interval (ISI, jitter 1800–2500 ms) consisted of a fixation cross. The baseline consisted of a centrally presented fixation star and was presented in five blocks evenly arranged across the task, each with a duration of 24.5 s. Subjects were instructed to indicate by right-hand button press whether the probe was part of the stimulus or not. Response button assignment (‘right/left’) was counterbalanced across participants. Stimulus delivery and response registration was controlled by Presentation (Neurobehavioral Systems Inc., Albany, CA, USA).

### Data Acquisition

EEG (impedance <20 kΩ [51]) was recorded from 60 scalp electrodes using MR-conditional caps (Easycap, Germany) simultaneously during fMRI scanning. EEG montage was based on a selection of 10–20 system positions [52]. Two additional electrodes were used to record eye movements (EOG) and 2 further electrodes were used to obtain the electrocardiogram (ECG). F1 served as recording reference, and F2 was the ground electrode. Data were sampled at 5 kHz (input range: 32 mV, bandpass filter: 0.1–250 Hz). FMRI data were acquired on a 3.0 T GE scanner with a T2*-sensitive EPI sequence (TR = 1.815 s; TE = 32 ms; FOV = 22 cm; image matrix = 64×64; voxel size  = 3.44×3.44×3.8 mm^3^; flip angle = 75°, 33 axial slices).

### Data Preprocessing

FMRI data were pre-processed and analyzed using SPM5 (Wellcome Department of Cognitive Neurology, London, UK). Images were realigned to those from the first volume to correct for head motion (exclusion criterion: motion >2 mm and/or translation >2° rotation). Images were normalized to the MNI standard brain template, resampled to 3 mm^3^ voxels and spatially smoothed (9 mm FWHM). For the EEG data, MR-gradient and ECG artifacts were removed by the average artifact subtraction method [53]. EEG data was digitally bandpass filtered (0.5–70 Hz, 24 dB/octave and 50 Hz Notch) and downsampled to 256 Hz. An infomax independent component analysis [54] was calculated to decompose the signal into EEG and artifact components. After excluding artifact-related components from the back projection, the EEG was transformed to the average reference [55].

### EEG Analysis

The spectral analysis focused on the last 2.5 s of the maintenance interval [56]. For each of these segments a Fast Fourier Transformation (FFT, Hanning window: 10%, frequency resolution of 0.25 Hz) was computed electrode-wise and averaged across segments separately for the two load conditions. The FFT was also calculated on baseline periods with the same segment length. All theta frequency measures were calculated as the band average between 5 and 7 Hz. Theta and alpha (8–13 Hz) power during the maintenance interval (i.e., load independent effects) were calculated relative to baseline power: (maintenance – baseline)/baseline *100. Load-dependent effects were calculated by: (load 5-load 2)/load 2 *100. For inference on group mean and group differences, t-statistics were calculated on the percentage data. T-thresholds of statistical significance were calculated for p<0.05 (t = 1.78) and p<0.01 (t = 2.56), uncorrected for multiple comparisons.

### GSP and GFS Regressor Construction

To estimate the total activity at a given frequency over the scalp, global spectral power (GSP) was calculated as the spatial root mean square across all FFT transformed scalp channels [10], [57]. Instead of averaging across trials, GSP estimates were extracted from each trial to form a vector reflecting trial-to-trial GSP variation.

Like GSP, GFS was calculated for each maintenance segment. GFS reflects the global amount of phase-locked (*zero-phase lag or instantaneous synchronization*) activity at a given frequency across the scalp [58]. Specifically, the GFS reflects the overall proportion of instantaneous synchronization of oscillations due to connectivity from spatially distinct (extended or separated) sources, along with contributions due to field spread of active sources. The GFS is independent of the recording reference and of implicit source models. This becomes apparent when looking at a sine-cosine diagram of a multichannel EEG, where the origin of the diagram corresponds to the recording reference. A change of the recording reference thus translates to a change of origin of the sine-cosine diagram, but the relative positions of the points representing the different electrodes remain the same. The shape of the cloud of these points thus does not change. Since GFS measures how round or elongated this cloud is, the location of the origin of the graph is not relevant, GFS is thus reference independent (see figure S1).

Briefly, estimation of the GFS begins with the frequency transformation of each epoch. For each frequency bin, the sine- and cosine values of all EEG channels are plotted in a 2D diagram, yielding one point per electrode. The shape of the resulting cloud of points indicates the amount of zero-phase lag synchronization across electrodes: If the cloud is elongated, the EEG at the given frequency is dominated by a common phase across all electrodes, whereas if the cloud is nearly round no such predominant phase is present. This geometrical explanation also clarifies that GFS is a pure measure of common phase independent of amplitude. To quantify the shape of the sine–cosine cloud, a 2D principal component analysis is computed. The resulting two eigenvalues (e1 and e2) are used to compute GFS as the ratio (abs(e1−e2)/(e1+e2)). GFS ranges from 0 (e1 = e2, no predominant phase and minimal synchronization) to 1 (e1 or e2 equal to 0, all electrodes phase-locked, maximal synchronization). Because GFS is a global index, no electrode-wise analysis can be calculated.

Both GSP and GSF values were calculated on artifact-free segments, linearly interpolated to preserve the total number of 64 trials, and averaged within the theta band. To explain covarying BOLD signal in−/decrease, the vectors reflecting trial-to-trial GSP and GFS variation were used for the parametric modulation of the maintenance interval in the EEG informed fMRI analysis (see below).

### Conventional fMRI Analysis

FMRI analysis at the individual level was performed using the general linear model. The design matrix included all 4 task intervals, the first three (encoding, maintenance, and probe) with a parametric modulation of the load condition. Only trials with a correct response were included; incorrect trials were modeled in a separate regressor. The standard hemodynamic response function was used as well as a standard high-pass filter (128 s cut-off) and an autoregressive model accounting for serial correlations. A second-level random effect analysis was performed to assess load-dependent (load 5– load 2) within- and between-group effects. Voxel-wise t-statistics were considered significant if p<0.01 (t >3.14), corrected for multiple comparisons using the false discovery rate (FDR) method [59]. Load-dependent group differences were reported at p<0.001 (t >3.37, cluster corrected at p<0.05, k = 31, http://afni.nimh.nih.gov/pub/dist/doc/manual/AlphaSim.pdf) as is common for fMRI group comparisons, since the results were not significantly different using a FDR corrected voxel threshold.

### EEG Informed fMRI Analysis

The EEG-informed fMRI analysis was run on separate models for GSP and GFS and included the theta (5–7 Hz) band regressor. The design matrices were identical to those described for the conventional fMRI analysis, but the parametric load-modulation of the maintenance interval was replaced by either the theta GSP or GFS regressors (model 1). To disentangle the effects of EEG trial-to-trial fluctuations from the load effects, we also ran the analysis with the load modulation as additional parametric modulator of the maintenance interval (model 2). Hence, the GLM consists of the following regressors: maintenance, maintenance load, theta EEG regressor (maintenance only), encoding, encoding load, probe, probe load, and ISI (model 2). The random effects analysis was also identical to that for the conventional fMRI analysis. Within- and between group-effects were considered significant at p<0.001 corrected, using a cluster size threshold (p<0.05, k = 31). Since we also wanted to study the interaction between EEG-BOLD signal correlation strength and performance, we additionally performed a GSP-BOLD and GFS-BOLD signal correlation analysis with load 5 response accuracy (no ceiling effect) as a covariate of no interest.

### Demographic and Behavioral Data Analysis

Percent correct responses (accuracy) and RT were estimated for all participants. Between-group differences were assessed by a repeated-measures ANOVA with the factor group (adults and children) and condition (load 2 and load 5). Individual mean GSP/GFS values were correlated to individual accuracy and RT (including both load conditions).

## Results

### Behavioral Effects

In general, adults performed better during the WM task than children. Adults showed significantly higher response accuracy during the task than children (adults: overall performance (%): 96.8, load 2∶97.2, load 5∶96.5; children: overall performance (%): 89.3, load 2∶94.8, load 5∶84.2). The group differences in response accuracy reached significance both for the overall performance and for the higher load condition (adults - children: overall performance: F_1,32_ = 8.5, t = 3.3, p = 0.003; load 5: t = 3.7, p<0.0001). In addition, adults responded significantly faster during the probe interval (adults: overall mean RT (ms): 1030, load 2∶954, load 5∶1107; children: overall mean RT (ms): 1353, load 2∶1274, load 5∶1440). The between-group differences in response time reached significance for both load conditions as well as for the mean RT over all conditions (adults - children: overall mean RT: F_1,32_ = 9.2, t = 4.2, p<0.0001; load 2: t = 3.8, p<0.001; load 5: t = 4.2, p<0.0001).

### EEG Effects

**Figure 1:**
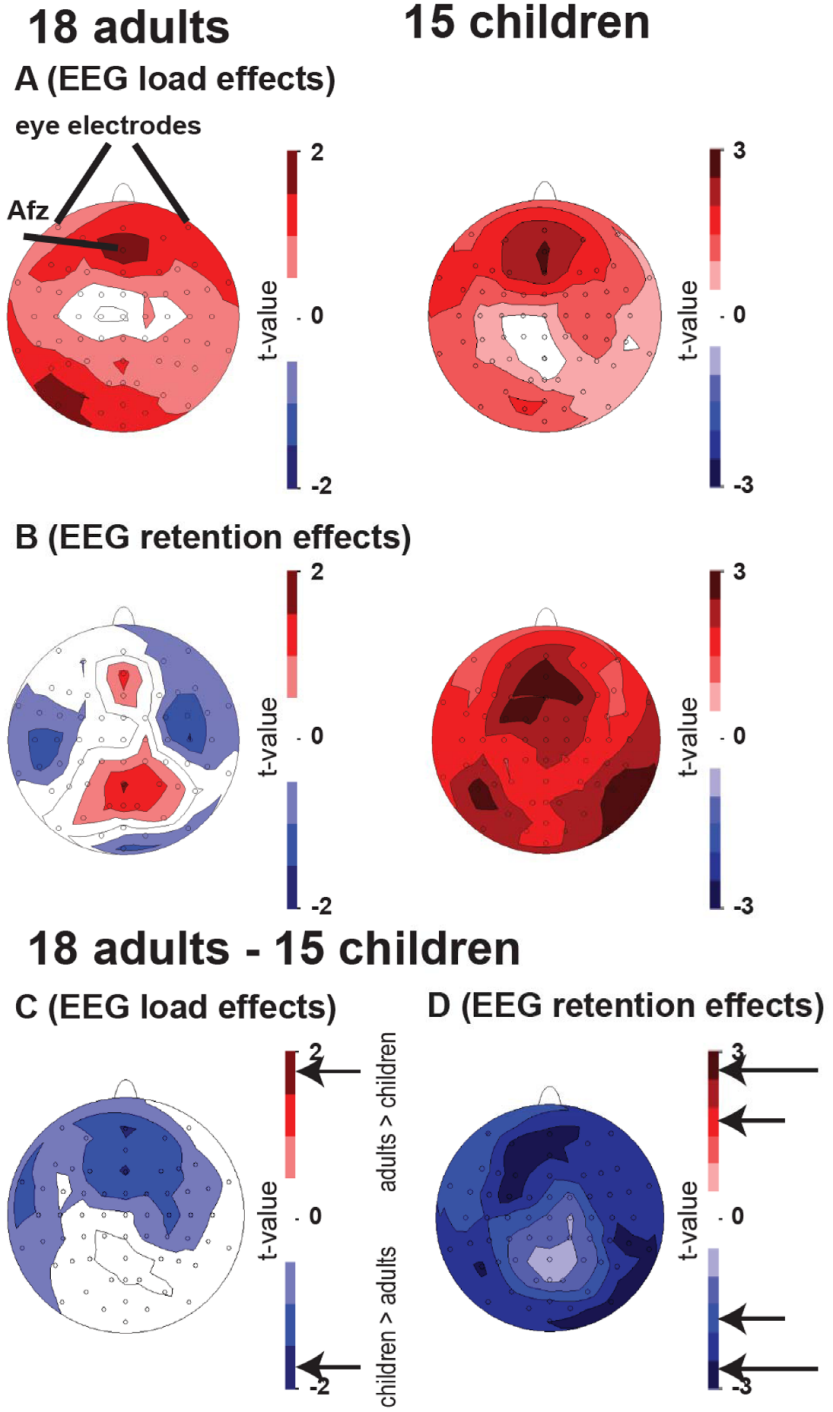
Topographical distribution of EEG working memory effects. For adults and children, load dependent and load independent theta EEG effects are shown in subfigures **A** and **B**, respectively. Group differences are shown in **C** and **D**. The short arrow indicates p<0.05 (t >1.69) and the long arrow indicates p<0.01 (t >2.44).

**Figure 2:**
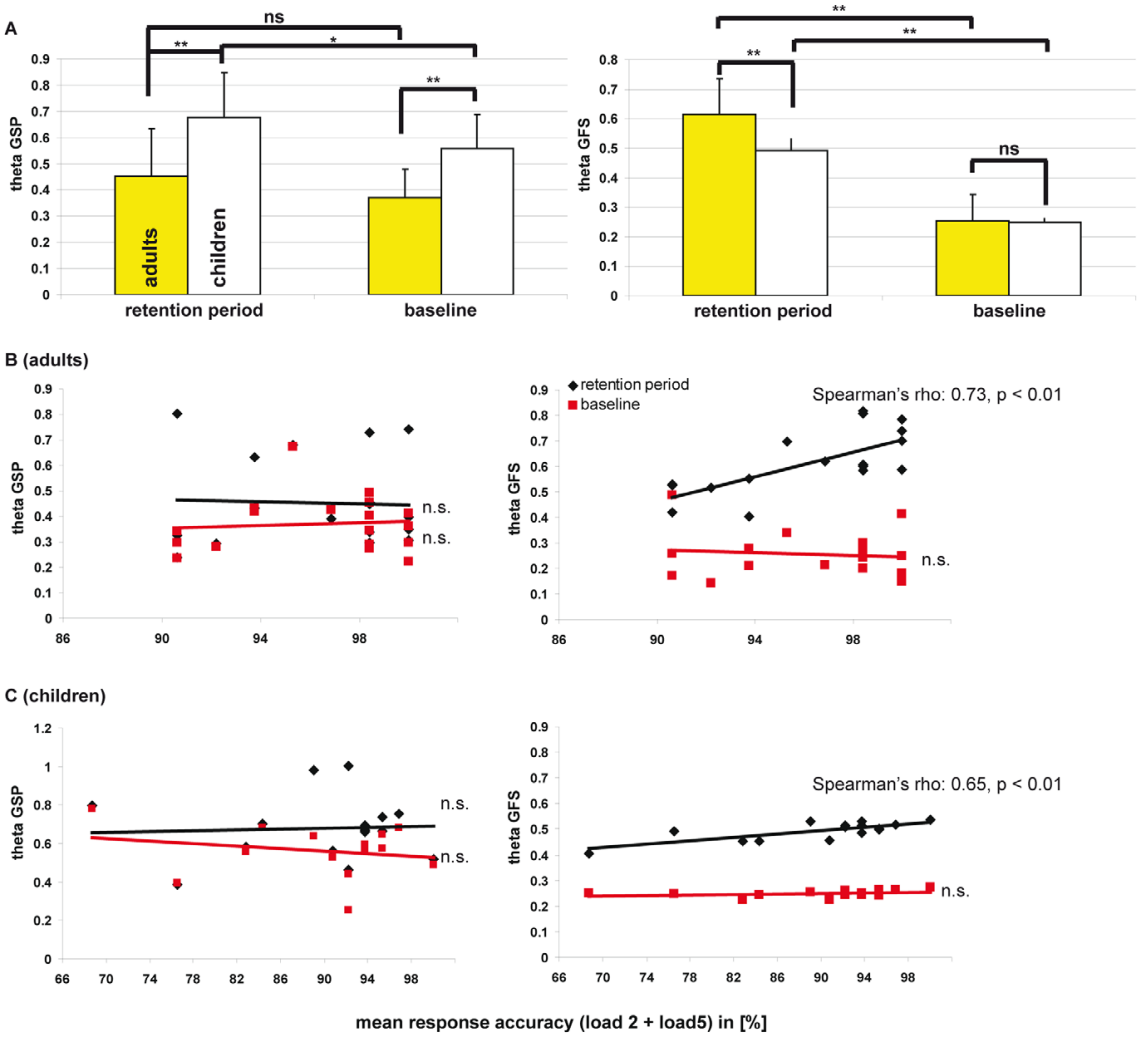
Mean theta EEG GSP and GFS along with GSP/GFS-response accuracy interactions. Results for GSP (maintenance and baseline interval) are shown in the left panels of **A**, **B**, and **C**, whereas results for the GFS are shown in the right panels of **A**, **B**, and **C**. (** indicates p<0.001, * indicates p<0.05. n.s.: non-significant.

For both groups, the topographic spectral analysis revealed significant load independent and load dependent power effects at frontal electrodes (Fig. 1A-B). Load independent and dependent effects were significantly stronger for children than for adults, particularly at frontal and right parietal electrodes (t >1.78 and t >2.56, Fig. 1C). Alpha power was manifested predominantly at parietal electrodes but load dependent and load independent effects did not differ between age groups. Therefore, EEG results for alpha are only shown in figure S2. A distributed source localization analysis revealed load-dependent theta effects for both groups at the anterior cingulate cortex (ACC) that were, however, only significant in children (figure S3). Theta GSP was lower for children than adults (0.45±0.18 vs. 0.68±0.17; p<0.0009, Fig. 2A, left panel) whereas theta GFS was higher for adults than for children during the maintenance interval (0.62±0.12 versus 0.49±0.04; p<0.0007, Fig. 2B, right panel). For children, theta GSP was significantly higher during the maintenance than during baseline interval (adults showed a trend, p<0.1). For both groups, theta GFS was significantly higher during the maintenance than during the baseline interval. A positive correlation was seen between mean response accuracy and theta GFS for the maintenance interval in both groups (Spearman’s rho_(adults)_: 0.73, p<0.01; Spearman’s rho_(children)_: 0.65, p<0.01), but not with baseline theta GFS (Fig. 2B/C, right panels). No significant correlation was observed between theta GSP/GFS and RT.

### FMRI Effects

**Figure 3:**
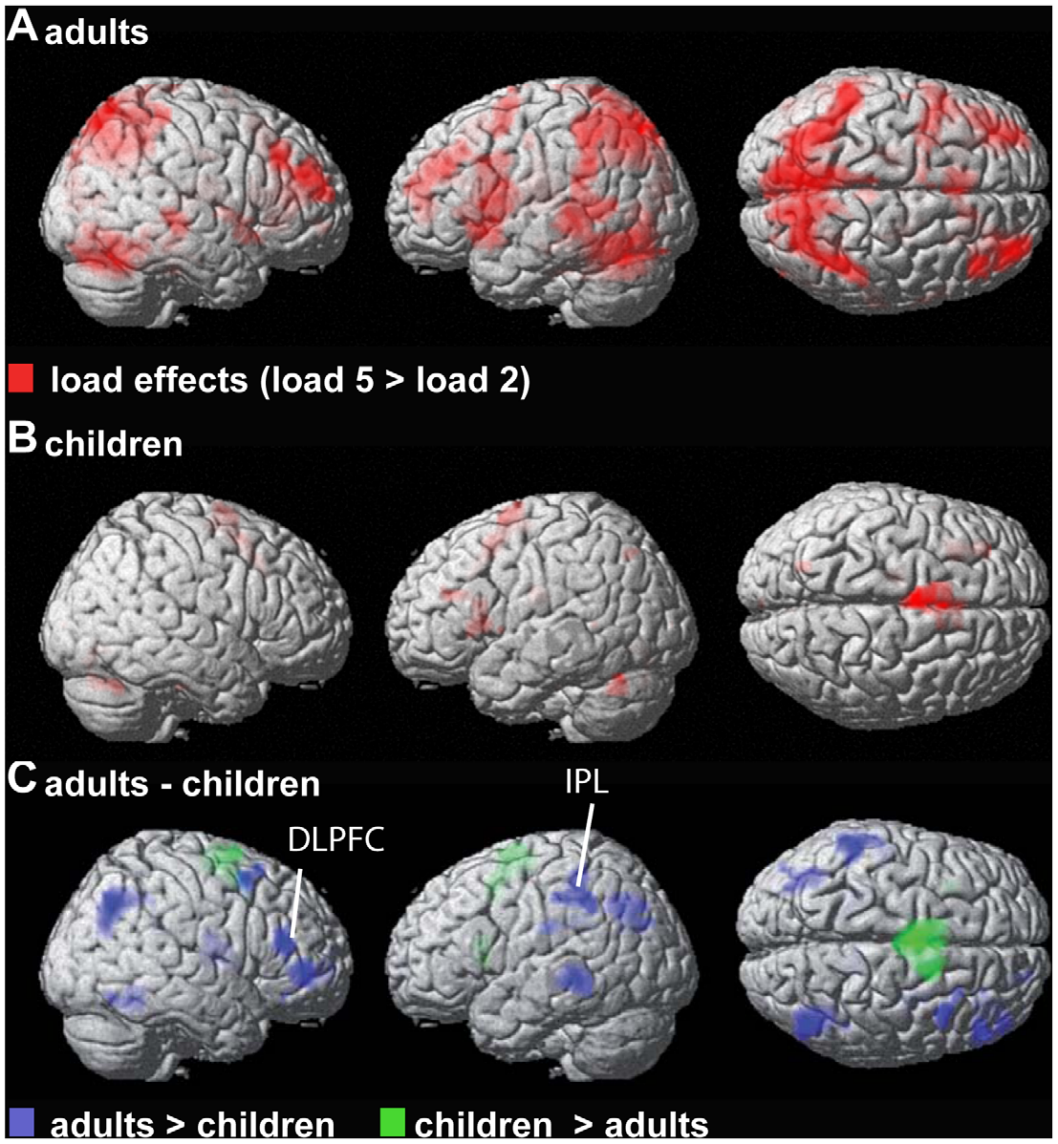
Load-dependent BOLD signal changes. Results are shown for adults **A**, children **B**, and for the group contrast: adults – children **C**. Within-group results are presented at p<0.01 (FDR corrected). Between-group results are shown at p<0.001 (uncorrected) with a cluster-correction of p<0.05. IPL: inferior parietal lobe; DLPFC: dorsolateral prefrontal cortex.

Load dependent fMRI activations were observed for both groups in similar regions but these load effects were stronger for adults (Table 1 and Fig. 3). Load-independent group results were stronger in adults than in children (not shown) but were not significant at p<0.01 (FDR corrected).

**Table 1:** Load dependent fMRI activation differences between adults and children.

adults > children				
region	BA	clustersize	t-value(peak voxel)	MNIx,y,z {mm}
R IPL	39/40	174	4.6	45 −63 42
L STG	22	82	4	−63 −39 −6
R MFG	10	186	4	39 42 0
L Precuneus	19	119	3.8	−36 −66 39
L Cingulate gyrus	31	46	3.7	−24 −42 30
R Insula	13	148	3.6	30 −9 15
R SFG	6	47	3.4	39 12 54
L IPL	40	93	3.4	−57 −45 39
R Cingulate gyrus		46	3.3	18 −42 27
R FG	37	38	3.2	54 −57 −18
R MPFC	10	36	3.2	18 54 2
**children > adults**				
**region**	**BA**	**cluster** **size**	**t-value** **(peak voxel)**	**MNI** **x,y,z {mm}**
L MPFC	6	127	6	−9 6 51
R SFG	6	106	3.8	12 9 54

Results are reported at a voxel threshold of p<0.001 (uncorrected) with a cluster-correction of p<0.05. R, right; L, left; IPL, inferior parietal lobe; STG, superior temporal gyrus; MFG, middle frontal gyrus; SFG, superior frontal gyrus; FG, fusiform gyrus; MPFC, medial prefrontal cortex.

### EEG-BOLD Signal Correlations

Regardless of age, correlations with the BOLD signal were exclusively negative for theta power (GSP) but exclusively positive for theta synchronization (GFS). Since we did not find significant load-(in)dependent alpha power differences between both group of subjects, results for alpha-BOLD signal correlations are not presented in this manuscript.

### GSP-BOLD Signal Correlations

**Figure 4:**
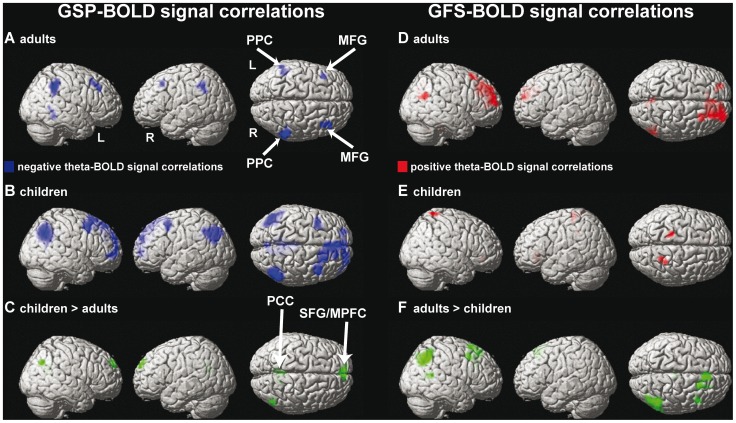
Theta GSP-BOLD and GFS-BOLD signal correlations. Within-group results for theta GSP-BOLD signal correlations are shown in **A**-**C**, while GFS-BOLD signal correlations are shown in **D**-**F**. Between-group comparisons are presented in **C** and **F**. For all subfigures, results are shown at p<0.001 (uncorrected), except for subfigure E (p<0.01, uncorrected). Abbreviations: PPC, posterior parietal lobe; MFG, middle frontal gyrus; MPFC, medial prefrontal cortex; PCC, posterior cingulate cortex, R, right hemisphere; L, left hemisphere.

For model 1 (without workload in the GLM), adults exhibited negative theta GSP-BOLD signal correlations bilaterally in the lateral PPC and middle frontal gyrus (MFG), as shown in Fig. 4A. Children exhibited negative GSP-BOLD signal correlations in a similar but more extended neuronal network than adults, including the PPC, precuneus, SFG, PCC, MFG, and MPFC as shown in Fig. 4B. The group comparison revealed significantly stronger negative GSP-BOLD signal correlations for children than adults (Fig. 4C) in the (right) PPC, PCC, MPFC, SFG, MFG, and angular gyrus (AG). No significant between-group differences were found in subcortical regions such as the thalamus. Model 2 (with workload in the GLM) yields very similar results, for adults and children, i.e. within-group results were not different (paired t-tests, all p>0.01, corrected for multiple comparisons) between both models. These results are presented in figure S4.

### GFS-BOLD Signal Correlations

For model 1, significant positive theta GFS-BOLD signal for adults were seen at the border of the SFG and MPFC, right precuneus, PCC, middle temporal gyrus (MTG), and cerebellum (Fig 4 D). At a lower statistical threshold (p<0.01, k = 133), correlations were also observed bilaterally in the posterior thalamus as well as in the nucleus caudatus. In children, correlations were positive and present at posterior and midline prefrontal structures but failed to reach significance (p>0.001, uncorrected, Fig. 4E). The group comparison revealed significant differences (adults > children) at the MPFC, SFG and MFG, AG, MTG, and cerebellum (Fig. 4F) but not in subcortical regions. For the GSP-BOLD signal correlation analysis, within-group results were again highly similar between model 1 and model 2 (paired t-tests, all p>0.01, corrected) as shown in figure S4.

## Discussion

The major findings of this study are the developmental changes observed for the separate EEG and fMRI measures as well as for both global EEG-fMRI correlations. Adults showed stronger load-dependent fMRI activity than children (Table 1), while children showed stronger load-dependent (and load-independent) FM-theta GSP than adults (Fig. 1). Both groups demonstrated exclusively negative theta GSP-BOLD signal correlations within the DMN, which were stronger in children (Fig. 4). In contrast to GSP, adults demonstrated stronger theta GFS and stronger positive theta GFS-BOLD signal correlations in fronto-parietal regions compared to children. Further, theta GFS but not GSP was positively linked to accuracy in both groups (Fig. 2).

### GSP

In humans, there is long-standing evidence that theta oscillations play a crucial role in the recruitment of brain circuits for the purpose of information processing [60], [61], [62]. Specifically, theta but also gamma (30–100 Hz) have been hypothesized to present a direct neural correlate of WM maintenance, possibly in cooperation with medial temporal lobe brain regions [5], [63], [64], [65], [66]. However, the precise role of theta oscillations during the development of cognitive functions is not yet fully understood. Our observation of a phasic event-related increased FM-theta power with load during the maintenance interval replicates previously published EEG/MEG work on WM during a similar task [5], [6], [7], [67]. Since rest activity was subtracted for both load conditions and age groups, task-related group differences (children > adults) cannot simply reflect the well-known increase of low frequency resting state power in children [18], [19], [68]. Although FM-theta power has been observed even in young children [69], [70], our results demonstrating stronger FM-theta power in school-aged children most likely reflect brain immaturity, as children performed significantly worse than adults. Further, we did not find any interaction between response accuracy and theta GSP, either for adults or for children, even if we selected the frontal electrode AFz, which shows a generally high WM modulation [6], [56]. This finding is consistent with the literature, as significant interactions between EEG power and accuracy during WM have been observed previously in alpha but not in the theta band [71]. Further, it has been argued that spectral power reflects local synchronization [22], as it measures synchronous cell activity under a given electrode. In line with this notion, children in our study demonstrate significantly higher local (GSP) but not global theta synchronization relative to adults (see below).

For both theta-GFP and theta-GFS there is an assumption that the same processes contribute to the variation in the signal in both children and adults during the WM task. In principle, the sources contributing to variation in the GFP and GFS could be markedly different between the two age groups, for instance due to incomplete maturation of frontal brain regions. However, this is unlikely given the similar topographies for adults and children (Fig. 1), because they point to a common task-related source in some medial prefrontal regions, such as the ACC. This is plausible, since our [10] and other groups [11] have shown in EEG-fMRI studies that theta power load effects are visible at the ACC in adults using a Sternberg WM task. Indeed, we could show through a distributed EEG source analysis that children and adults showed load effects in prefrontal locations, i.e. in the dorsal (children) and ventral (adults) ACC (figure S3). Thus, we would argue that in both groups the same underlying sources contribute to variation in the EEG feature of interest.

### GFS

The global synchronization measure GFS is defined as the proportion of EEG activity oscillating with a common phase at a given frequency across channels, irrespective of location. The calculation of GFS is based on several assumptions, particularly that the amplitude of phase-locked oscillations is mediated through networks of cortico–cortical and thalamocortical connections. There are several physiological studies in humans and animals as well as modeling studies emphasizing the relevance of instantaneous, i.e. zero-phase lag synchrony [57], [72], [73], [74], [75], [76]. High GFS values indicate strong functional binding while low values suggest a state of more or less permanent disconnection. As multiple EEG sources are presumably active at the same time in the brain, a high GFS value is further assumed to indicate that all active generators are interacting as a single, spatially-distributed global process. This property also distinguishes GFS from coherence values, which are spatially but not time specific; both measures can be further interpreted depending on the frequency band considered. In a recent study it has been shown that resting theta GFS differentiated Parkisonian patients from healthy controls [77], demonstrating its sensitivity to detect subtle changes in theta band activity. Further, it has been demonstrated that resting GFS increases during development for the alpha band [78]. It is known that theta (and alpha) scalp EEG coherence (non-phase locked synchronization) between medial frontal- and parietal pairs increases during development [79], [80], [81] and is modulated during WM in adults [35], [82], [83], [84]. Our result of decreased theta GFS in children relative to adults is in line with these developmental findings regarding resting state coherence, and also with previous studies reporting lower theta [22] and alpha [38], [85] phase locking in children compared to adults during an auditory oddball task. We found that the strength of theta GFS (but not GSP) correlated positively with response accuracy in adults and children. Thus, our results partly extend previous findings that individuals with a good memory performance show increased EEG theta (and alpha) phase coupling during recognition [86]. In contrast, RT was not correlated to theta GSP, in line with results from a recent EEG-fMRI study investigating WM [11]. The observed link between theta GFS and behavior suggests that theta GFS reflects an increased whole brain network dynamic, especially in adults as we found stronger theta GFS for adults compared to children. The lower theta GFS in children might indicate reduced (global) phase locked activity, and thus point to a different mechanism of neural coding in maintaining items during WM performance. Specifically, temporal coding or synaptic plasticity, i.e. a precise timing of neural spikes, is relevant for the coding of sensory stimulation [87]. At the macroscopic level, oscillatory brain activity such as hippocampal theta bursts can facilitate temporal coding [88]. Interpreting such task-related GFS development in terms of increased temporal coding is also consistent with the suggestion that adults but not children may predominantly use temporal coding during auditory tasks, based on developmental increases of different EEG synchronization measures [22]. More generally, these results are in agreement with the notion that temporal coding emerges at lager stages of development and learning [89]. Therefore, our finding of increased GFS in adults most probably reflects neural maturation, since non-neural maturation due to lower skull conductivity (i.e., reduced scalp amplitudes and increased field spread) in adults would lead to increased coherence and synchronization regardless of the condition [90].

### FMRI Effects

Load dependent activity was stronger in adults than children in regions that have been related to verbal WM tasks [16], [41], [42], [91], [92]. Thus, we could replicate multiple developmental fMRI or optical topography studies investigating spatial [47], [48] or verbal WM [16], [17], [43], [44], [45], [46], [49]. For example, two of these studies used variants of the Sternberg task and found weaker prefrontal and parietal activation in children [16] or adolescents [43] compared to adults, similar to our results. Those studies found also stronger load-dependent cerebellar activations in adults, which are consistent with the results from the present study, since we also observe stronger load-dependent cerebellar BOLD signal changes in adults relative to children at a lower statistical threshold.

### GSP-BOLD Signal Correlations

The normal cellular mechanism underlying EEG (MEG) signal requires the metabolism of glucose and an abundant supply of oxygen, Thus, the measured EEG signal is assumed to be closely related to the underlying spatio-temporal pattern of metabolism in the normal human brain [93], [94], [95]. Recently we showed in a nearly identical group of subjects that GSP-BOLD signal correlations are not significantly different between children and adults for eyes open and eyes closed resting conditions [50], irrespective of the examined frequency band. The only coupling differences (i.e., stronger for adults) were seen in the thalamus, indicating that resting EEG-BOLD signal correlations can identify weak developmental effects. In this study, we found predominantly negative theta GSP-BOLD signal correlations in a set of regions that together form the DMN [96]. Negative theta-BOLD signal correlations have previously been observed during resting states [3] and during simultaneous [10], [11] and serial [97] EEG-fMRI studies of WM in adults. Our results extend these studies as we found a correlation pattern within the DMN not only in adults but even stronger in children. Thus, in contrast to resting EEG-fMRI studies, we suggest that significant developmental effects during cognition can be assessed not only by EEG and fMRI alone but also by simultaneous EEG-fMRI measurements. However, one difference relative to our previously published results was that we could not detect correlations with the MPFC or the PCC in the group of adults at the pre-selected statistical threshold [10]. Even if the EEG-BOLD signal analysis was performed with FM-theta power (i.e., absolute EEG power was derived from electrode AFz), which usually shows strong load effects [6], [7], [56], theta-BOLD signal correlations remained weak at these locations (figure S5). This additional analysis therefore demonstrates that the choice of the GSP for the fMRI correlation analysis provides comparable results to those using selected electrodes or components in this instance [11].

### GFS-BOLD Signal Correlations

A recent study in adults reported bidirectional resting alpha GFS-BOLD signal correlations in regions of the DMN, suggesting that in addition to the spectral dynamics of the EEG also the spatial-temporal organization is an important aspect to consider in the characterization and understanding of brain processes [57]. From previous EEG studies it is known that prefrontal and temporo-parietal regions show a strong coherence in the theta frequency range during WM retrieval or maintenance [82], [98]. Although several EEG/MEG studies observed (phase-lagged) synchronized long range oscillations during cognitive processing in adults [82], [99], [100], [101], which are weaker in children than in adults [22], it was still unknown whether synchronization is linked to the BOLD signal during cognitive operations in children and adults. Our study reveals, that the group comparisons of theta GFS-BOLD signal correlations encompass a few brain regions of the DMN, namely the MPFC and PPC, which demonstrate stronger GFS-BOLD signal correlations in adults than in children. These results show that theta GFS can capture not only developmental effects but also requires, in contrast to the GSP, an elevated level of oxygen (see subsequent paragraph). Since GFS captures zero-phase lag coherence, GFS could in principle not only reflect neural synchronization due to functional long range connectivity, but also apparent synchronization due to field spread/volume conduction [102]. However, empirically, the neuronal activity measured by EEG appears to be closely reflected in fMRI signal changes [103]. Our findings are in line with this notion and inconsistent with pure volume conduction effects which could not influence the BOLD signal, since we found that higher theta GFS was paralleled by higher BOLD activity in adults and that lower theta GFS was paralleled by lower BOLD activity in children (see Limitations section below). Further, we would argue that behavioral parameters cannot explain the differences between age-related theta GFS-BOLD and GSP-BOLD signal correlations, as most of the correlations were preserved even after correction for response accuracy under high workload (figure S6).

### Possible Physiological Mechanisms Underlying Task-related EEG-BOLD Signal Correlations During Cognitive Development

Several MRI-, task- and resting state functional connectivity fMRI studies have revealed that the DMN (especially fronto-parietal long-range connections) is not fully developed in children and that white matter maturation is positively correlated with the degree of cortical activation in the frontal and parietal DMN regions [14], [15], [92], [104], [105]. Although the observed link between maturation and cortical activation is in agreement with our findings -i.e. adults showed stronger load-dependent fMRI activations than children- the notion that the DMN is not fully developed in children is not. Specifically, the stronger EEG-BOLD signal correlations seen in the DMN in children relative to adults do not appear consistent with the assumption that this network is not fully developed (assuming that EEG-BOLD signal correlations capture functional connectivity to a certain degree). Recently, it has been shown in epileptic children that a markedly abnormal EEG with high signal amplitude and disorganized delta/theta activity is related to reduced resting-state functional connectivity (i.e. abnormally low phase-locked slow spontaneous fluctuations of the BOLD signal), which was restored after successful corpus callosum surgery [106]. This result clearly demonstrates the interplay between low frequency activity and functional and anatomical connectivity. Since the children examined in the present study are healthy, the stronger theta and theta-BOLD signal correlations do not reveal anything about the integrity of anatomical or functional connectivity but rather indicate that frontal theta EEG scalp power is accompanied by decreased oxygen consumption during cognitive processing. It has been theorized that the inverse relation between theta and the DMN activity in adults indicates that theta power is a marker for task preparedness, as the DMN becomes less active during a task [3]. As children show even stronger theta-BOLD correlations than adults, we would suggest that children might require a higher level of task-preparedness to adequately perform on the WM task, which can be achieved by a stronger suppression of a task-irrelevant network, namely the DMN. On the other hand, it is known that structural brain development and particularly synaptic pruning is heavily associated with gray matter reduction and myelination. Further, the grey matter reduction is often assumed to underlie functional development, such as decreasing low frequency resting EEG power and increasing functional connectivity with increasing age. A basic question is whether this physiological mechanism also contributes to the development of EEG-BOLD coupling during cognitive brain functions such as WM. It has recently been hypothesized that the adolescent decline in EEG power reflects a widespread brain reorganization which is driven by pruning [107], [108]. The significantly stronger theta BOLD signal correlation observed in children might therefore not only be related to an increased level of task-preparedness but may also result from an oversupply of non-functional neurons which are eliminated or “pruned” during brain development. Hence, stronger task-related EEG power and theta BOLD signal correlations might be explained by the observation that neural activity is more competitive during development, stabilizing coincident and weakening non-coincident inputs [109], [110], [111]. This might also explain why theta GFS-BOLD signal correlations are weaker in children than in adults, as the overshoot of synapses could cause diminished neuronal synchronization, which could be manifested in this study as lower GFS values in children compared to adults. As mentioned above, theta-BOLD signal correlations appear to be not only a marker for rest [3], [112] and cognition [8], [9], [10], [11], [97] in adults but also a marker for cognitive development. However, developmental changes in theta-BOLD correlations during cognition might not necessarily be expected across the full time period of brain development (see hypothesis 2), as we recently observed that broad-band (including theta) resting-state EEG-BOLD signal correlations are indistinguishable between adolescents and adults [112]. Taken with our previous results this finding indicates that the physiological coupling matures earlier and is therefore insensitive to developmental effects in adolescence which can be observed for the EEG and the fMRI alone. Nevertheless, future studies could examine the question of whether or not EEG-BOLD signal correlations differ between adolescents and adults during cognitive task operations.

### Limitations

GFS indexes the proportion of the (in this case theta band) EEG signal that can be explained by a single phase (i.e., instantaneous synchronization) over all electrodes. Instantaneous synchronization captures some specific and important, but certainly not all physiological aspects of synchronization due to functional connectivity. In addition, volume conduction may represent a confounding factor when interpreting zero-phase lag synchronization between electrodes, since the measure of GFS is not capable of un-mixing signals from different sources. Thus, for example the method does not distinguish between the activity of a single source, and the activity of several sources oscillating at the same phase, as both cases always lead to a GFS value of 1. However, it is important to note that volume conduction is immediate and therefore cannot itself produce any phase differences at the sensor level (except of course the 180 degree phase reversal for the opposite poles of a source). So if there such phase differences are present, there must have been more than one source active and these sources cannot have oscillated at the same phase. In general, given that we have an extended sensor array with the capability to detect the potential of relevant sources, we maintain that GFS measures the relative predominance of oscillations of a certain phase at the level of sources, while no information about the location of these sources is necessary. Volume conduction would present a major problem in the assessment of localization, and a reasonable separation of the effects of volume conduction and physiological zero-lag synchronization is only possible if the scalp fields produced by the potentially interacting sources are sufficiently uncorrelated. However, no pure non-invasive measure of instantaneous synchronization between distinct sources exists because all potential, current source density and MEG measures are characterized by volume conduction or field spread [113], and all source estimates require a-priori assumptions about source distributions or spread (smoothness, extent etc). Most importantly, we maintain that the instantaneous synchronization measured by GFS is useful and important because it is sensitive to physiologically meaningful zero-lag synchronized oscillations between separated sources. The problem of volume conduction can be avoided by considering only the imaginary part of the phase relations [102]. This is conceptually very different from instantaneous synchronization, and has other implications on the interpretation of the data. For example, synchronization measures based only on lagged correlations are completely unaffected by field spread, yet those measures do by definition also exclude instantaneous, physiologically meaningful synchronization between distinct sources on which we focus here. Further, synchronization computed from source estimates would remove some confounds due to field spread but requires (often implicit) assumptions about source spread.

### Conclusion

In summary, we demonstrate that WM related EEG-BOLD recordings detect specific developmental effects in the EEG-fMRI coupling strength in some regions of the DMN. Second, theta power and global field synchronization show an opposite coupling with the BOLD signal in both adults and children. From the power-BOLD signal correlation analysis we conclude that a task-irrelevant (i.e., DMN) network has to be suppressed during cognitive processing, especially in children, as evidenced by the negative correlation between theta power and the BOLD signal within the DMN. From the synchronization-BOLD signal correlation analysis we conclude that theta whole-scalp synchronization is positively coupled to the BOLD signal. As the coupling is stronger in adults than in children, we further conclude that that this coupling reflects a marker for cognitive development.

## Supporting Information

Figure S1 **Reference independence of the GFS measure.** A 19 channel EEG was recomputed to three different references; average reference, Fp1 and O1. All channels were then frequency transformed using the FFT, retaining the cosine (real) and sine (imaginary) parts. These values were plotted as black diamonds in the three graphs; the reference electrode (where available) is shown as red diamond and by definition at the origin of the graph. It becomes apparent that the change of the reference implies a mere shift of the data in reference to the origin of the coordinate system, while the relative positions among the points representing the electrodes remain unchanged. GFS is computed as the ratio of the norms of the first and the second principal components. These principal component vectors are displayed in magenta. The change of reference changes their position in the coordinate system in the same way as for the electrodes, but the length of the principal component vectors remains unchanged. Since only the length of these vectors enters the computation of the GFS value, it is not affected by any change of the reference electrode.
(TIF)

Figure S2 **Topographical distribution of theta and alpha EEG effects.**
(TIF)

Figure S3 **Distributed EEG source localization results for load-dependent effects (load 5– load 2) in the theta band (5–7 Hz).** Standardized low resolution brain electromagnetic tomography (sLORETA [114]) was used to localize the generators of the scalp EEG power spectra for the load-dependent contrast for adults (left panel) and children (right panel). The sLORETA solution space is restricted to the cortical grey matter in the digitized MNI atlas with a total of 6239 voxels at 5 mm spatial resolution [114]. A spatial over-smoothing of 10^−4^ was chosen for the LORETA transformation matrix. Since sLORETA explicitly takes into account that scalp electric potentials are determined up to an arbitrary additive constant, the final sLORETA solution is independent of the electrical reference used. Tomographic sLORETA images were calculated corresponding to the estimated neuronal generators of brain activity [115] for the theta band, using the same frequency band width as those for the spectral analysis. sLORETA images were statistically compared through multiple voxel-by-voxel comparisons (i.e., corrected p-value) using a common non-parametric test for functional brain imaging [116] and were plotted onto a standard MRI template, as described in detail elsewhere [114]. The significance threshold was based on a permutation test with 5000 permutations. Results reached only significance for children (p<0.05, t = 4.6).
(TIF)

Figure S4 **GSP-BOLD and GFS-BOLD signal correlations results with maintenance workload included as a regressor in the GLM (model 2).** Note that there were no significant within-group differences for both types of correlation analyses if model 2 was compared to model 1 (i.e., the workload was not modeled as a regressor in the GLM, Fig. 4).
(TIF)

Figure S5 **Comparison between theta EEG AFz-BOLD- and GSP-BOLD signal correlations.** Results for adults are shown in **A** and **C**, and for children in **B** and **D**. Significant differences between AFz-BOLD signal and GSP-BOLD signal correlations were only visible at an unconventional statistical threshold of p<0.005 (uncorrected, **E-F**). Red colors denote AFz-BOLD signal correlations > GSP-BOLD signal correlations, green colors denote GSP-BOLD signal correlations > AFz-BOLD signal correlations.
(TIF)

Figure S6 **Theta EEG GSP/GFS-BOLD signal coupling results with and without response accuracy as covariate of no interest.** **A** and **C**: without load 5 accuracy included, **B** and **D**: with load 5 accuracy as a covariate of no interest.
(TIF)
