# Supplementary figures and images for: Developmental Changes of BOLD Signal Correlations with Global Human EEG Power and Synchronization during Working Memory
Source: PLoS One. 2012 Jul 6;7(7):e39447. doi: 10.1371/journal.pone.0039447 (PMC3391196; doi:10.1371/journal.pone.0039447)

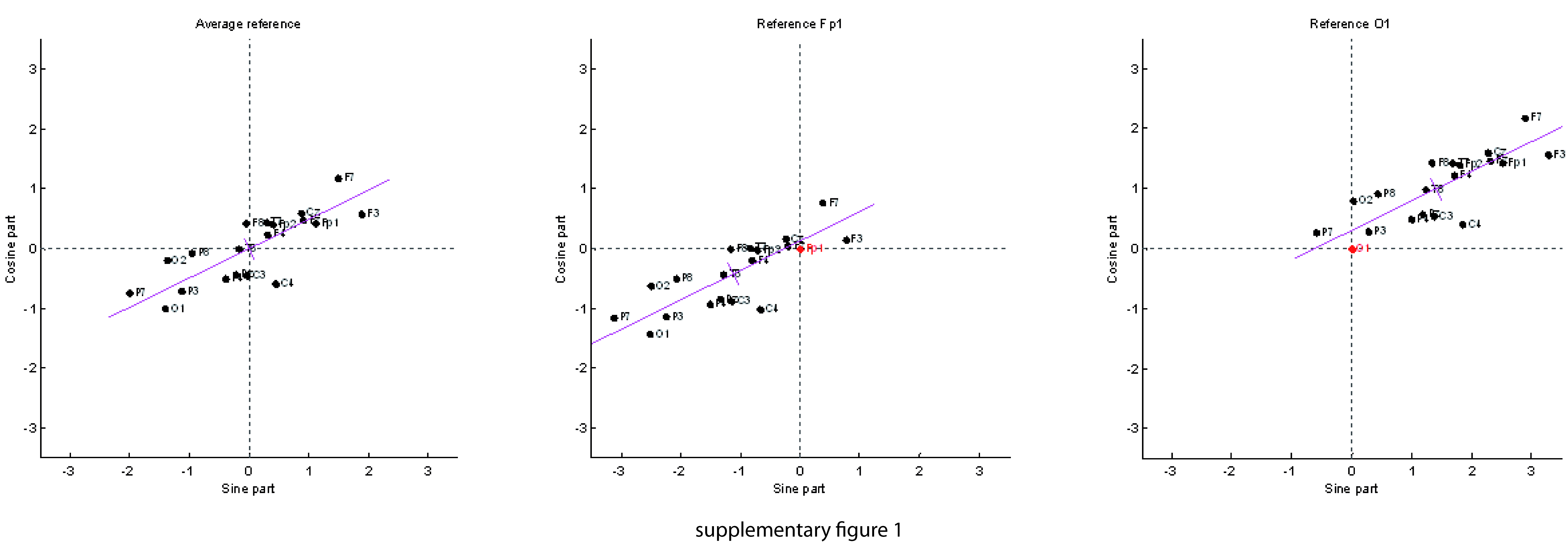

Supplement: Figure S1 — Reference independence of the GFS measure. A 19 channel EEG was recomputed to three different references; average reference, Fp1 and O1. All channels were then frequency transformed using the FFT, retaining the cosine (real) and sine (imaginary) parts. These values were plotted as black diamonds in the three graphs; the reference electrode (where available) is shown as red diamond and by definition at the origin of the graph. It becomes apparent that the change of the reference implies a mere shift of the data in reference to the origin of the coordinate system, while the relative positions among the points representing the electrodes remain unchanged. GFS is computed as the ratio of the norms of the first and the second principal components. These principal component vectors are displayed in magenta. The change of reference changes their position in the coordinate system in the same way as for the electrodes, but the length of the principal component vectors remains unchanged. Since only the length of these vectors enters the computation of the GFS value, it is not affected by any change of the reference electrode. (TIF) [file pone.0039447.s001.tif]

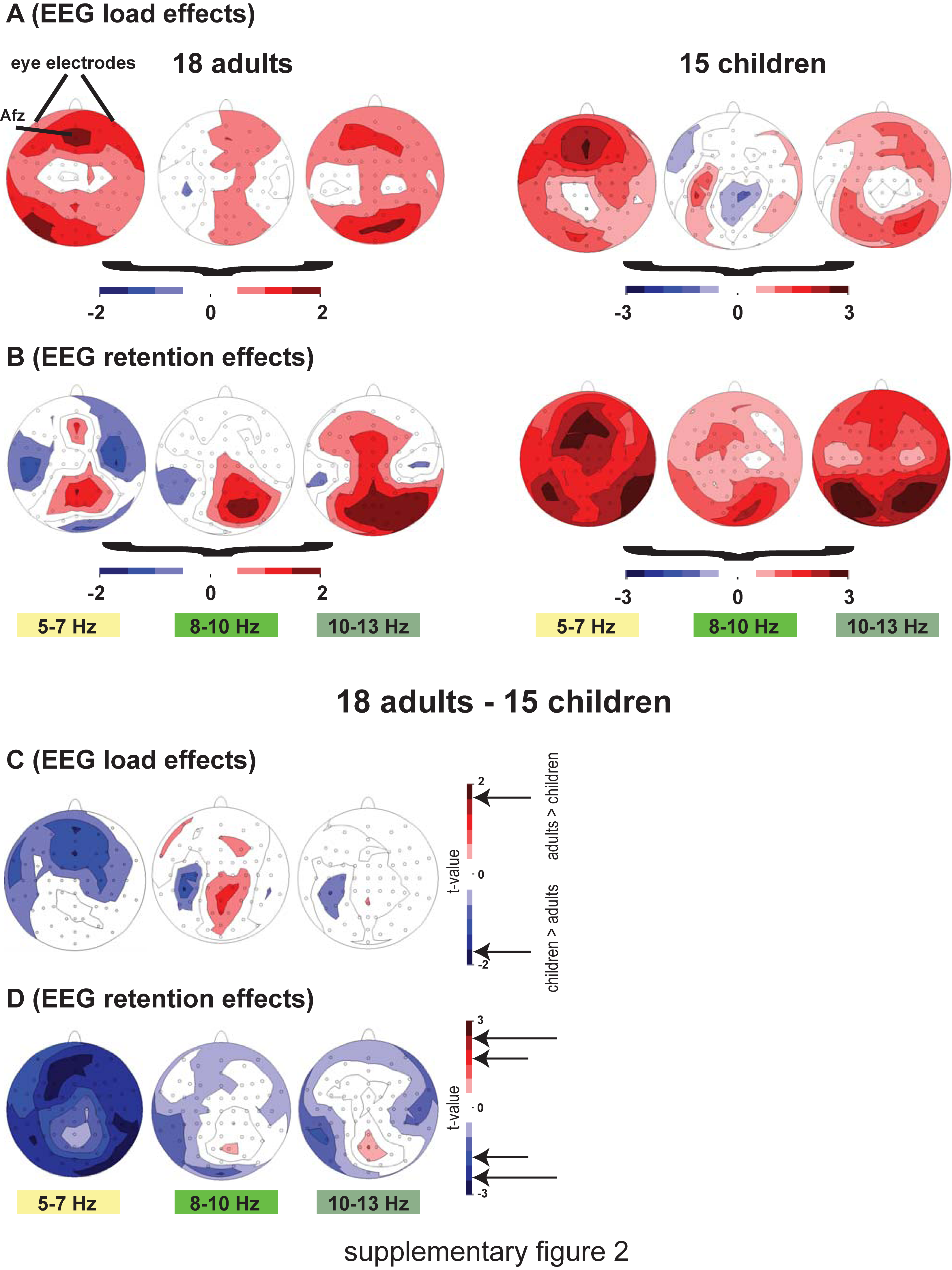

Supplement: Figure S2 — Topographical distribution of theta and alpha EEG effects. (TIF) [file pone.0039447.s002.tif]

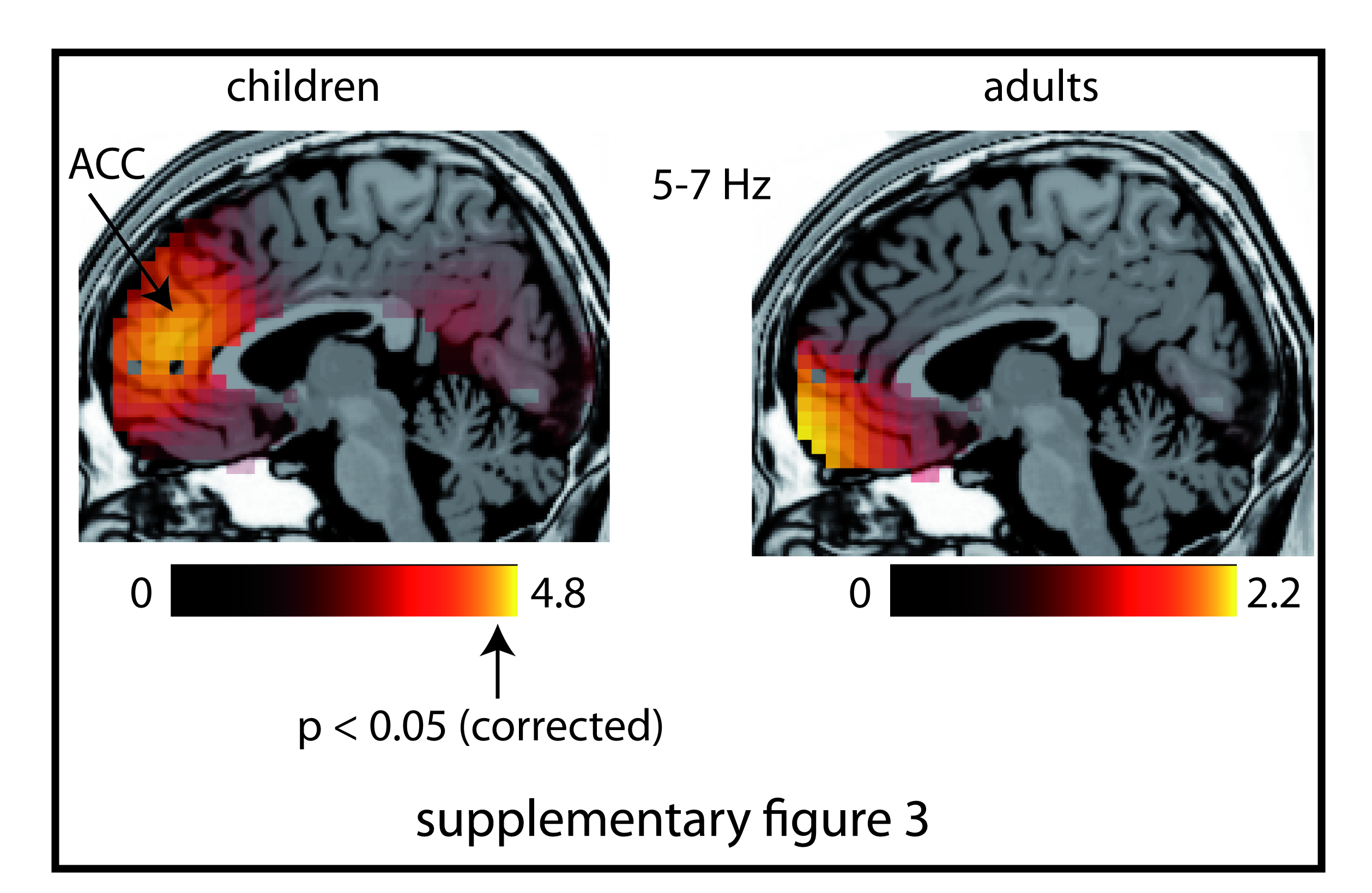

Supplement: Figure S3 — Distributed EEG source localization results for load-dependent effects (load 5– load 2) in the theta band (5–7 Hz). Standardized low resolution brain electromagnetic tomography (sLORETA [114]) was used to localize the generators of the scalp EEG power spectra for the load-dependent contrast for adults (left panel) and children (right panel). The sLORETA solution space is restricted to the cortical grey matter in the digitized MNI atlas with a total of 6239 voxels at 5 mm spatial resolution [114]. A spatial over-smoothing of 10−4 was chosen for the LORETA transformation matrix. Since sLORETA explicitly takes into account that scalp electric potentials are determined up to an arbitrary additive constant, the final sLORETA solution is independent of the electrical reference used. Tomographic sLORETA images were calculated corresponding to the estimated neuronal generators of brain activity [115] for the theta band, using the same frequency band width as those for the spectral analysis. sLORETA images were statistically compared through multiple voxel-by-voxel comparisons (i.e., corrected p-value) using a common non-parametric test for functional brain imaging [116] and were plotted onto a standard MRI template, as described in detail elsewhere [114]. The significance threshold was based on a permutation test with 5000 permutations. Results reached only significance for children (p<0.05, t = 4.6). (TIF) [file pone.0039447.s003.tif]

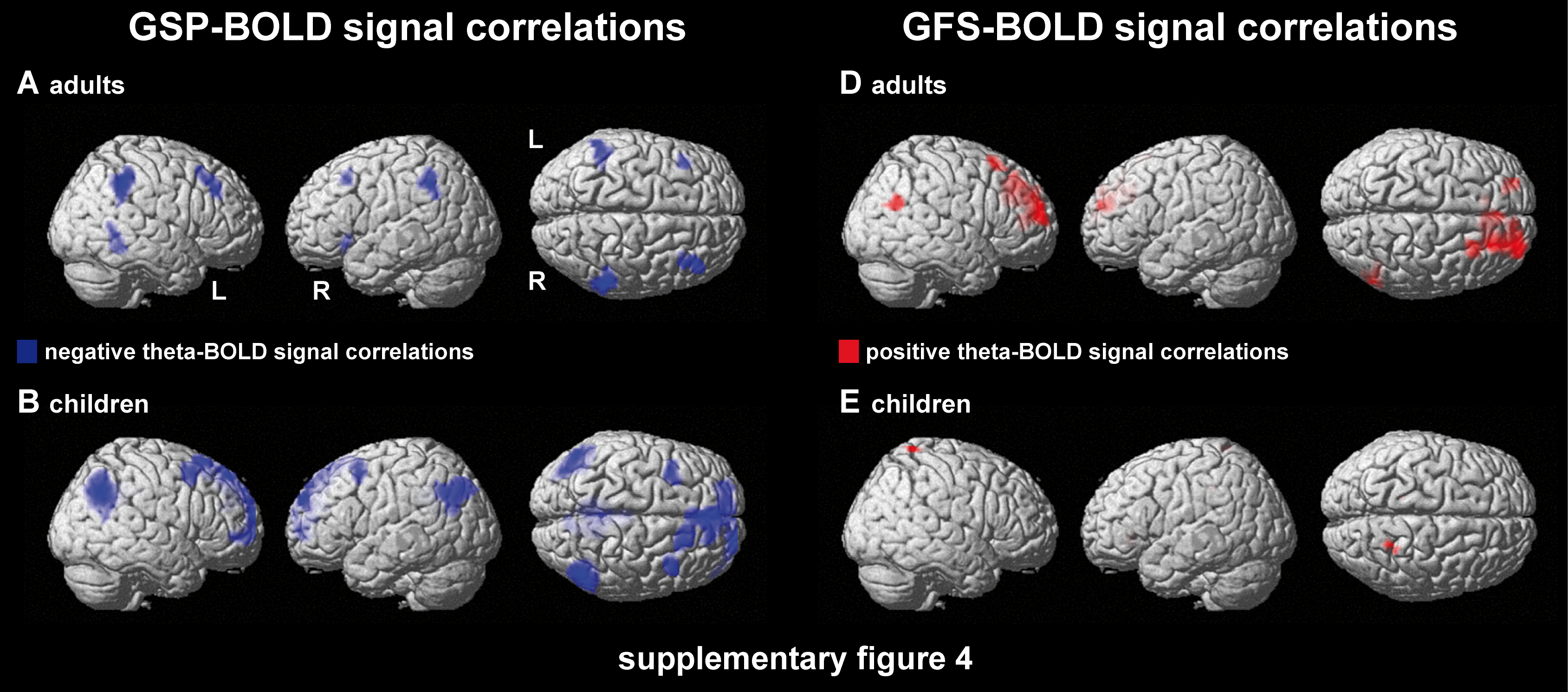

Supplement: Figure S4 — GSP-BOLD and GFS-BOLD signal correlations results with maintenance workload included as a regressor in the GLM (model 2). Note that there were no significant within-group differences for both types of correlation analyses if model 2 was compared to model 1 (i.e., the workload was not modeled as a regressor in the GLM, Fig. 4). (TIF) [file pone.0039447.s004.tif]

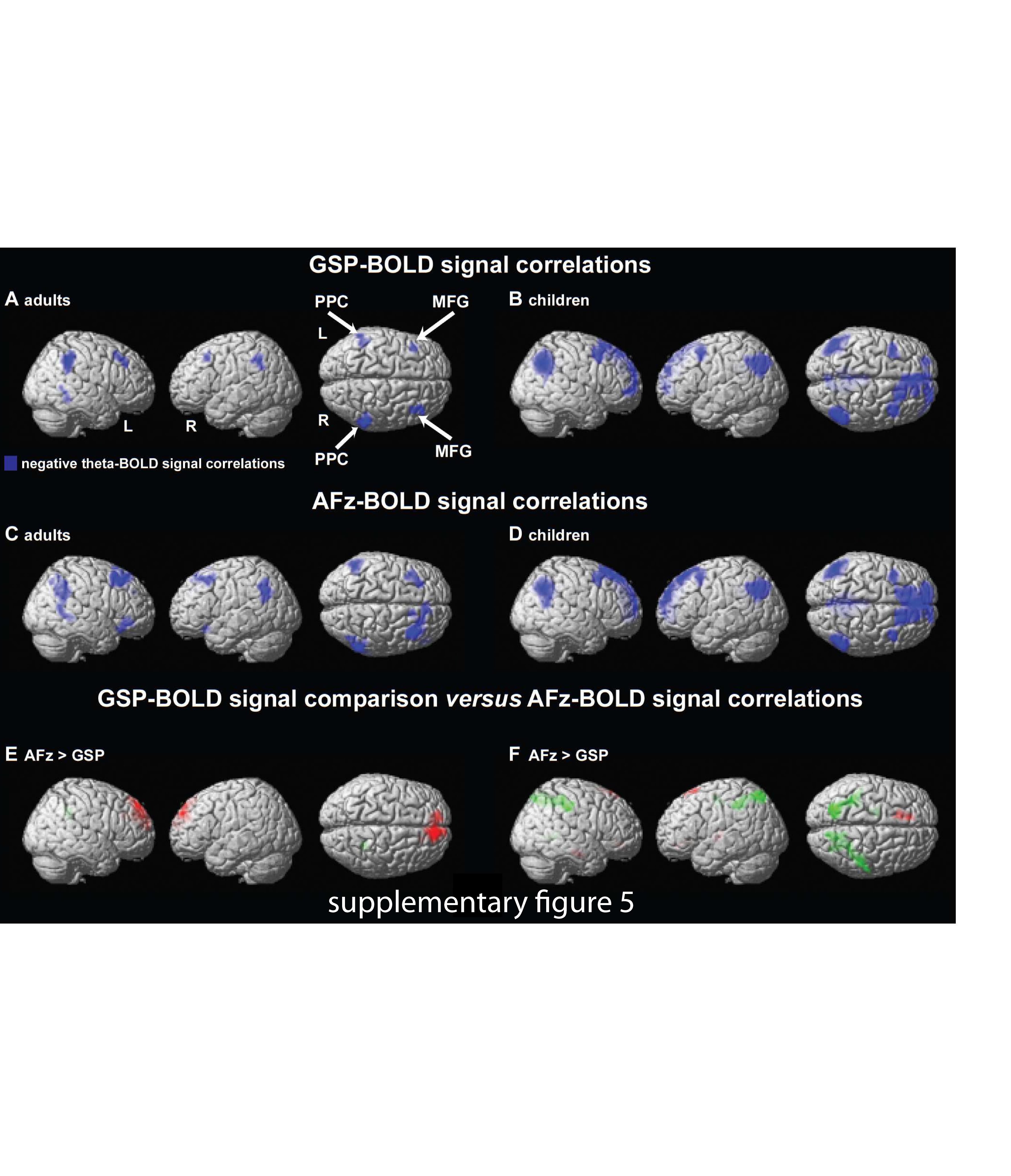

Supplement: Figure S5 — Comparison between theta EEG AFz-BOLD- and GSP-BOLD signal correlations. Results for adults are shown in A and C, and for children in B and D. Significant differences between AFz-BOLD signal and GSP-BOLD signal correlations were only visible at an unconventional statistical threshold of p<0.005 (uncorrected, E-F). Red colors denote AFz-BOLD signal correlations > GSP-BOLD signal correlations, green colors denote GSP-BOLD signal correlations > AFz-BOLD signal correlations. (TIF) [file pone.0039447.s005.tif]

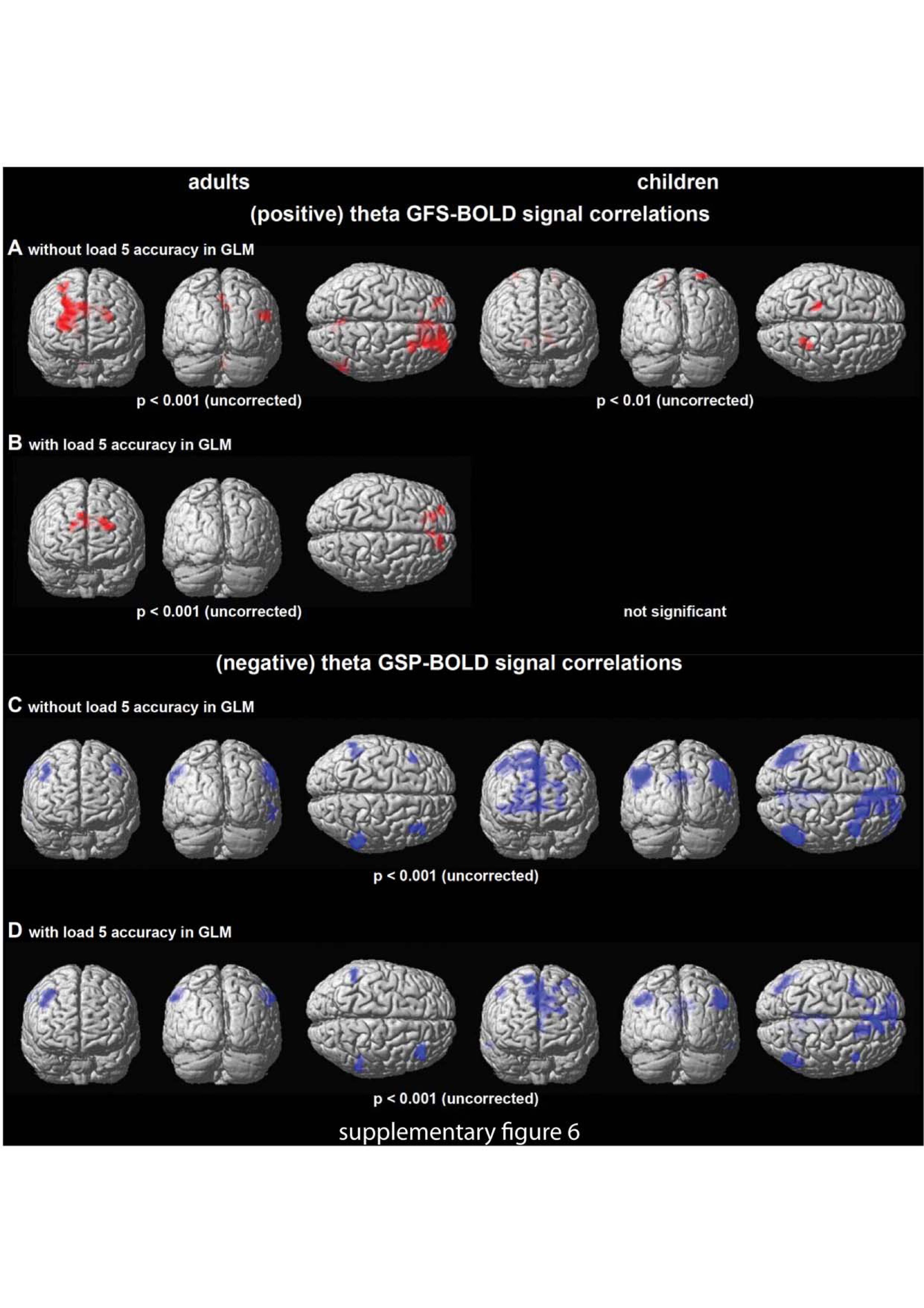

Supplement: Figure S6 — Theta EEG GSP/GFS-BOLD signal coupling results with and without response accuracy as covariate of no interest. A and C: without load 5 accuracy included, B and D: with load 5 accuracy as a covariate of no interest. (TIF) [file pone.0039447.s006.tif]
